# Supplementary material for: Impact of Anticoagulation and Sample Processing on the Quantification of Human Blood-Derived microRNA Signatures
Source: Cells. 2020 Aug 18;9(8):1915. doi: 10.3390/cells9081915 (PMC7464075; doi:10.3390/cells9081915)
Supplement: Supplementary file 1 [file cells-09-01915-s001.pdf]

Supplemental Material and Methods

Supplemental information regarding the miRNA panel

miR-197, miR150 and **miR223** are among the most highly expressed miRNAs in platelets and platelet microparticles and are significantly downregulated upon dual anti-platelet therapy (aspirin/dipyridamole or clopidogrel) in patients with carotid atherosclerosis [1, 2]. Additionally, plasma levels of **miR-223** are decreased in patients with essential thrombocytopenia [3]. **miR-23a** is highly abundant in platelets [4], but does neither correlate with the degree of platelet activation nor with clopidogrel resistance [5].

Plasma levels of **miR-191** were found to positively correlate with C-reactive protein and pro-inflammatory cytokines in patients with diabetes mellitus type 2 [6]. Circulating **miR-320** was significantly upregulated in patients with deep vein thrombosis [7]. **miR-24** levels is unique for leukocytes and plasma levels of miR-24 were decreased in patients with abdominal aortic aneurysm [8]. Circulating **miR-21** plasma levels were found to be positively associated with recurrent venous thromboembolism [9] and were increased in plasma samples contaminated by residual platelets [10].

miR-27a was identified as a positive regulator of inflammation [11] and is upregulated in macrophages upon exposure to LPS [12]. **miRNA-126** is highly expressed by endothelial cells, endothelial cell apoptotic bodies and platelets. **miRNA-126** plays an important role in the regulate vascular integrity, angiogenesis, and wound repair [13, 14] and decreased miR-126 plasma levels are associated with the development of type 2 diabetes [15]. Moreover, miR-126 is reduced in patients with antiplatelet therapy [1]. **miR-28** was significantly increased in the plasma of patients with active chronic inflammatory bowel disease [16] **miR-451** is highly expressed by erythrocytes and acts as a negative regulator of fatty acid-induced inflammation[17]

Literature:

1. Willeit, P., et al., *Circulating microRNAs as novel biomarkers for platelet activation.* Circ Res, 2013. **112**(4): p. 595-600.
2. Carino, A., et al., *Modulation of Circulating MicroRNAs Levels during the Switch from Clopidogrel to Ticagrelor.* Biomed Res Int, 2016. **2016**: p. 3968206.
3. Tran, J.Q.D., et al., *Platelet microRNA expression and association with platelet maturity and function in patients with essential thrombocythemia.* Platelets, 2020. **31**(3): p. 365-372.
4. Bao, H., et al., *Platelet-derived microparticles promote endothelial cell proliferation in hypertension via miR-142-3p.* FASEB J, 2018. **32**(7): p. 3912-3923.
5. Chen, S., et al., *Expression of miRNA-26a in platelets is associated with clopidogrel resistance following coronary stenting.* Exp Ther Med, 2016. **12**(1): p. 518-524.

6. Dangwal, S., et al., *Impairment of Wound Healing in Patients With Type 2 Diabetes Mellitus Influences Circulating MicroRNA Patterns via Inflammatory Cytokines.* Arterioscler Thromb Vasc Biol, 2015. **35**(6): p. 1480-8.
7. Jiang, Z., et al., *Combination of Circulating miRNA-320a/b and D-Dimer Improves Diagnostic Accuracy in Deep Vein Thrombosis Patients.* Med Sci Monit, 2018. **24**: p. 2031-2037.
8. Maegdefessel, L., et al., *miR-24 limits aortic vascular inflammation and murine abdominal aneurysm development.* Nat Commun, 2014. **5**: p. 5214.
9. Wang, X., et al., *Association of recurrent venous thromboembolism and circulating microRNAs.* Clin Epigenetics, 2019. **11**(1): p. 28.
10. Mitchell, A.J., et al., *Platelets confound the measurement of extracellular miRNA in archived plasma.* Sci Rep, 2016. **6**: p. 32651.
11. Xie, N., et al., *miR-27a regulates inflammatory response of macrophages by targeting IL-10.* J Immunol, 2014. **193**(1): p. 327-334.
12. Wang, D., et al., *MiR-27-3p regulates TLR2/4-dependent mouse alveolar macrophage activation by targetting PPARgamma.* Clin Sci (Lond), 2018. **132**(9): p. 943-958.
13. Fish, J.E., et al., *miR-126 regulates angiogenic signaling and vascular integrity.* Dev Cell, 2008. **15**(2): p. 272-84.
14. Wang, S., et al., *The endothelial-specific microRNA miR-126 governs vascular integrity and angiogenesis.* Dev Cell, 2008. **15**(2): p. 261-71.
15. Zampetaki, A., et al., *Plasma microRNA profiling reveals loss of endothelial miR-126 and other microRNAs in type 2 diabetes.* Circ Res, 2010. **107**(6): p. 810-7.
16. Wu, F., et al., *Peripheral blood microRNAs distinguish active ulcerative colitis and Crohn's disease.* Inflamm Bowel Dis, 2011. **17**(1): p. 241-50.
17. Hur, W., et al., *Downregulation of microRNA-451 in non-alcoholic steatohepatitis inhibits fatty acid-induced proinflammatory cytokine production through the AMPK/AKT pathway.* Int J Biochem Cell Biol, 2015. **64**: p. 265-76.

Detailed statistical analysis

Influence of platelet activation of miRNA abundance: To determine whether and to which extent platelet activation might influence the measured abundance of miRNAs, a hierarchical regression approach was applied for each miRNA separately. For this purpose, data of all experimental settings were used, i.e. of all anticoagulants, all time points etc. First, five binary dummy variables were generated, representing the six volunteers. In a first block (model 1), only the dummy variables were entered. In a second block, PF4 (log-transformed) was added (model 2). The dummy variables were used to account for the dependency of values obtained from each of the volunteers. The p-value corresponding to the F change between model 1 and 2 was used to test whether logPF4 explained variance independent of the volunteers, the R^2 change (=R^2 of model 1 subtracted from R^2 of model 2) indicates which proportion of miRNA variance can be explained by PF4 after adjustment for the volunteers. To test whether PF4, TSP1 and sCD62P (i.e. platelet activation in general) predict miRNA abundance, they were entered after log-transformation in model 2 instead of PF4 alone, i.e. two different versions of model 2 were built: The first to test whether PF4 alone predicts miRNA abundance, the second to test whether PF4, TSP1 and sCD62P taken together predict miRNA abundance. To visualize to what extent the models predict miRNA abundance, unstandardized predicted values were saved for models 1 and 2. The difference of these models represents the predicted

values that were only attributable to the platelet activation markers were generated. These values were plotted against the measured miRNA values; the R^2 value of these two variables equals the R^2 change between model 1 and 2. The given R^2-values are the proportion of variance that can be explained by platelet activation parameters. The associated p-values refer to the hypothesis that platelet parameters predict miRNA abundance.

Influence of time on miRNA abundance: Referring to the question whether miRNA abundance could also be measured later in time after blood sampling, Pearson correlation coefficients between the Cq-values of several time points were calculated, specifically between 0.5/2 h, 0.5/6 h, and 0.5/24 h, assuming that immediate sample processing (i.e. 0.5 h) resembles most closely the real *in vivo* values. This was done separately for each miRNA in CTAD anti-coagulated blood processed at 4°C and at room temperature. Under the assumption that the effect of temperature and time on the measured miRNA species are also similar on other miRNAs that were not quantified in this study, results might be generalized. To estimate a general correlation, the 12 miRNA species were dummy coded by 11 binary factors. Then the partial linear correlation coefficient was calculated between the time points indicated above, adjusting for miRNA species. To visualize the uncertainty, 95% confidence intervals were calculated by Bias corrected accelerated bootstrapping with 1000 samples.

It has to be mentioned that the above described regression models and partial correlation coefficients were not subject to adjustment for multiple testing due to the exploratory character of this study. Results need to be interpreted accordingly. The generalizability of the findings, specifically conclusions regarding how miRNA samples should be processed *in general*, relies on the assumption that the six blood donors are representative for the general population, and that the chosen miRNAs are representative of others concerning their influenceability by sample preparation. All statistical analyses were performed using IBM SPSS Statistics 26, graphs were generated by Graphpad Prism 8.3.0. p-values < 0.05 were considered statistically significant.

Supplemental Results

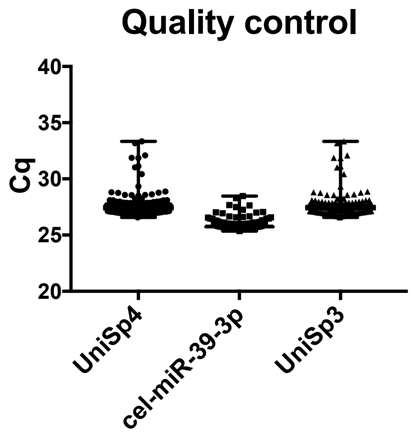

Supplemental Figure 1: Quality control of miRNA analysis was performed by multiple spike-in controls. UniSp4 was added during RNA isolation, cel-miR-39-3p during cDNA synthesis and UniSp3 during qPCR.

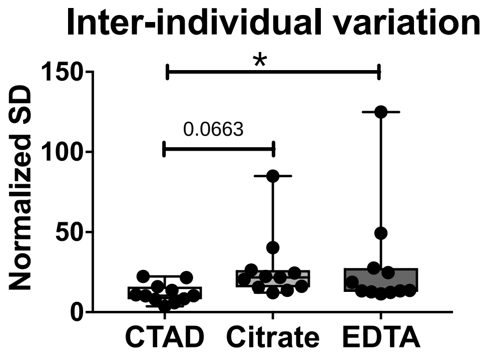

Supplemental Figure 2: Inter-individual variation of plasma miRNA levels was affected by the choice of anticoagulant. Standard derivation of normalized miRNA levels was compared between CTAD, citrate and EDTA plasma samples. Significant differences were determined using one-way ANOVA and are depicted as *p<0.05.

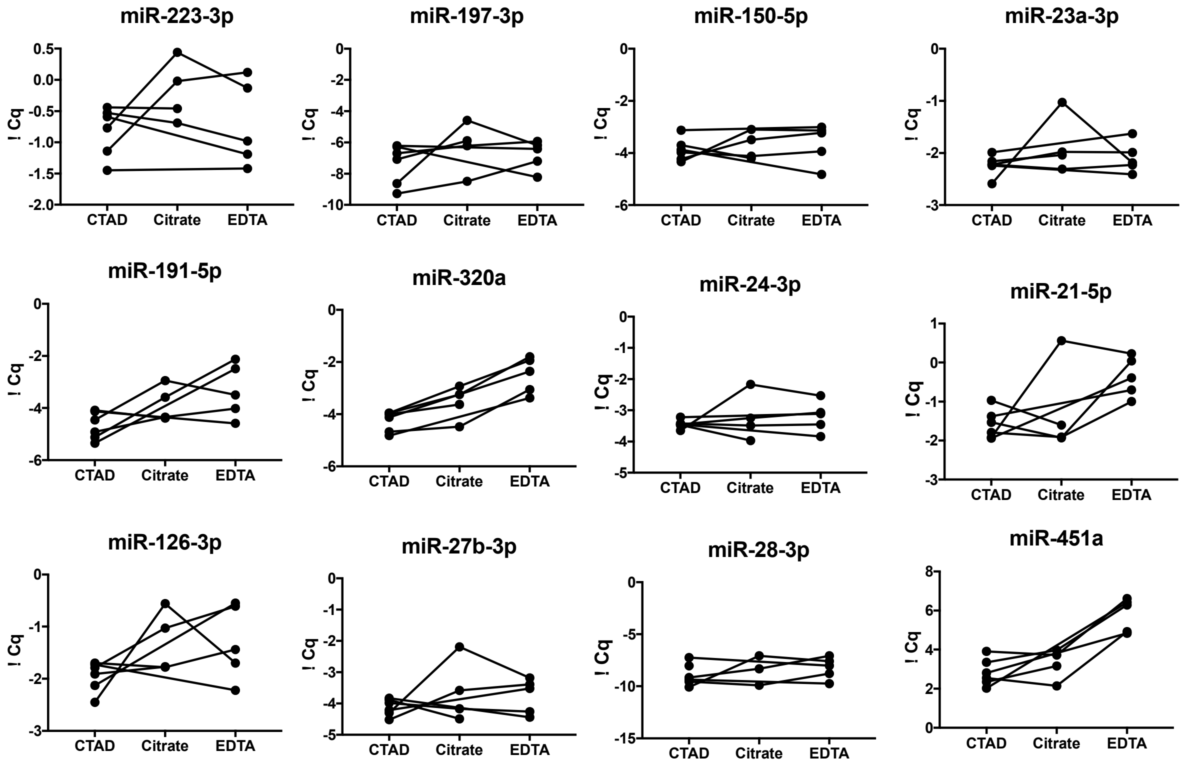

Supplemental Figure 3: Subject-specific analysis of plasma miRNA levels. Data from Figure 1 are displayed in a pairwise manner.

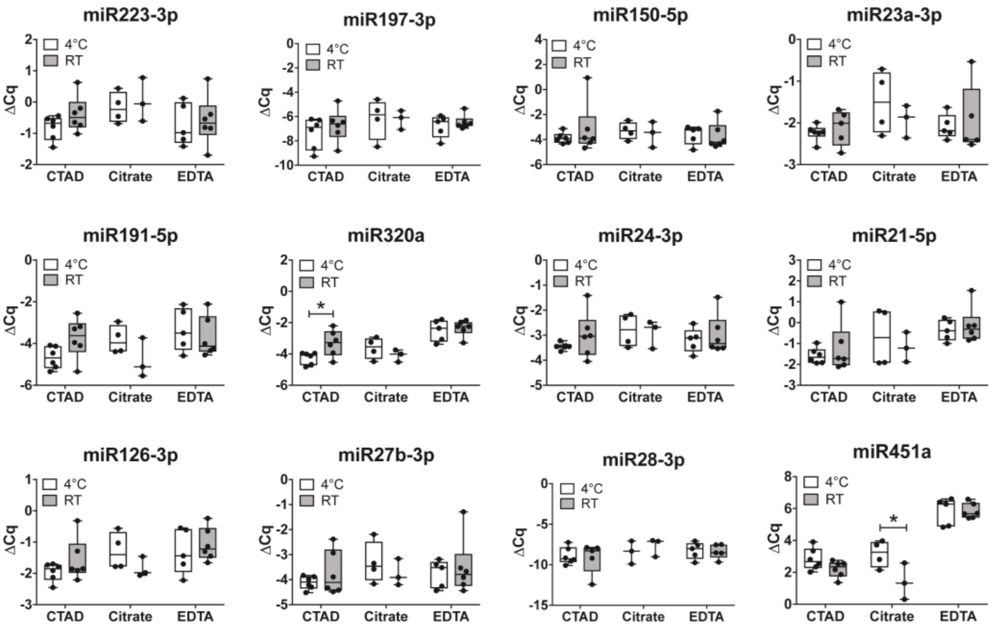

Supplemental Figure 4: Effect of temperature on plasma miRNA levels. Blood from six healthy donors was anticoagulated with CTAD, citrate or EDTA and plasma prepared within 30 min. ΔCq levels of platelet, cardiovascular and inflammatory miRNAs were determined for

samples either stored at 4°C or RT. Significant differences were determined using two-way ANOVA and Sidak's multiple comparison test and were depicted as *p<0.05.

Supplemental Tables

	n=6
**Age (yrs)**	28 (26-30)
**Females**	3 (50%)
**Males**	3 (50%)
**Caucasian Ethnicity**	6 (100%)

Supplemental Table 1: Demographic variables for study participants. Values represent mean values with range. Relative numbers (as percent of total) can be found in parenthesis.

CTAD	Time	126-3p	223-3p	191-5p	24-3p	21-5p	28-3p	27b-3p	320a	150-5p	197-3p	23a-3p	451a
	2 h	-0,709	-0,501	0,566	-0,848	0,179	-0,645	-0,195	0,355	-0,001	0,435	-0,249	0,479
	6 h	0,161	0,674	0,677	-0,014	0,313	-0,378	0,214	-0,009	0,14	-0,764	0,322	-0,619
	24 h	0,673	0,397	0,665	-0,655	0,619	0,016	-0,063	0,612	-0,252	-0,233	-0,078	-0,195

Citrate	Time	126-3p	223-3p	191-5p	24-3p	21-5p	28-3p	27b-3p	320a	150-5p	197-3p	23a-3p	451a
	2 h	-0,055	-0,143	0,099	0,314	0,382	0,988	0,321	0,344	0,703	0,359	0,712	0,655
	6 h	-0,034	-0,099	-0,557	-0,066	0,302	0,881	-0,113	-0,144	0,217	0,273	0,128	-0,358
	24 h	0,459	0,034	0,629	0,203	0,18	0,688	0,434	0,464	-0,134	0,248	-0,071	0,56

EDTA	Time	126-3p	223-3p	191-5p	24-3p	21-5p	28-3p	27b-3p	320a	150-5p	197-3p	23a-3p	451a
	2 h	0,533	0,517	0,252	0,367	0,227	-0,385	0,33	0,401	0,439	0,142	0,591	0,357
	6 h	-0,739	-0,97	-0,795	-0,925	-0,953	-0,876	-0,786	-0,892	-0,515	0,275	-0,771	-0,806
	24 h	0,083	-0,001	-0,235	-0,559	-0,69	0,091	0,164	-0,365	-0,623	0,555	0,358	-0,398

Supplemental Table 2: Pearson correlation coefficient of miRNAs stored at 4 °C. Pearson correlation coefficients between timepoint 0.5 h and 2 h, 6 h and 24 h timepoints for all miRNAs after storage at 4°C. Pearson correlation coefficients (r) of +1 indicates that the respective time point gives the same result as the 0.5 h time point; values of 0 would indicate that the values

of that time point are unrelated to the values obtained at 0.5 h, and negative values indicate inverse values.

	4 °C	RT
miR-223-3p	0,0006	<0,0001
miR-197-3p	0,193	0,0001
miR-150-5p	<0,0001	0,0063
miR-23a-3p	0,1217	0,0003
miR-191-5p	<0,0001	<0,0001
miR-320a	0,0002	0,0002
miR-24-3p	0,0034	0,0002
miR-21-5p	0,4298	0,031
miR-126-3p	0,3321	0,0729
miR-27b-3p	0,2308	0,0058
miR-28-3p	0,213	0,5735
miR-451a	0,0036	0,0008

Supplemental Table 3: Effect of storage time on the level of plasma miRNAs. Data represent p-values calculated for the variable time by two-way ANOVA.

CTAD	Time	126-3p	223-3p	191-5p	24-3p	21-5p	28-3p	27b-3p	320a	150-5p	197-3p	23a-3p	451a
	2 h	0,779	0,299	0,691	0,39	0,039	0,478	0,704	0,571	0,914	-0,143	0,543	0,408
	6 h	0,721	0,535	0,582	0,595	0,236	0,606	0,736	-0,501	0,767	0,772	-0,199	0,148
	24 h	-0,312	-0,723	-0,439	-0,259	-0,137	-0,623	-0,665	-0,208	0,595	-0,466	0,957	0,481

Citrate	Time (h)	126-3p	223-3p	191-5p	24-3p	21-5p	28-3p	27b-3p	320a	150-5p	197-3p	23a-3p	451a
	2 h	0,052	-0,236	0,132	0,142	0,164	0,171	0,069	0,725	0,599	0,231	0,362	0,848
	6 h	0,235	-0,429	0,017	0,292	0,023	-0,217	0,1	0,649	0,232	-0,078	0,018	-0,241
	24 h	0,414	0,22	0,295	0,098	0,126	-0,339	0,165	-0,074	0,301	0,356	0,279	-0,411

EDTA	Time (h)	126-3p	223-3p	191-5p	24-3p	21-5p	28-3p	27b-3p	320a	150-5p	197-3p	23a-3p	451a
	2 h	0,879	0,901	0,872	0,984	0,938	0,439	0,973	0,473	0,956	0,916	0,997	0,622

6 h	0,671	-0,136	0,385	-0,182	0,842	0,32	-0,115	- 0,651	-0,169	-0,114	-0,368	0,239
24 h	0,932	-0,159	0,52	0,353	0,689	0,031	-0,135	0,113	0,71	-0,519	0,29	- 0,035

Supplemental Table 4: Pearson correlation coefficient of miRNAs stored at RT. Pearson correlation coefficients between timepoint 0.5 h and 2 h, 6 h and 24 h timepoints for all miRNAs after storage at RT. Pearson correlation coefficients (r) of +1 indicates that the respective time point gives the same result as the 0.5 h time point; values of 0 would indicate that the values of that time point are unrelated to the values obtained at 0.5 h, and negative values indicate inverse values.
